# Prevalence, Genotypic Characteristics and Antibiotic Resistance of *Listeria monocytogenes* From Retail Foods in Bulk in Zhejiang Province, China

**DOI:** 10.3389/fmicb.2019.01710

**Published:** 2019-07-25

**Authors:** Yunyi Zhang, Shilei Dong, Honghu Chen, Jiancai Chen, Junyan Zhang, Zhen Zhang, Yong Yang, Ziyan Xu, Li Zhan, Lingling Mei

**Affiliations:** ^1^Department of Microbiology, Zhejiang Provincial Center for Disease Control and Prevention, Hangzhou, China; ^2^Department of Clinical Laboratory, Zhejiang Hospital, Hangzhou, China; ^3^Department of Biotechnology, Wenzhou Medical University, Wenzhou, China

**Keywords:** *Listeria monocytogenes*, prevalence, retail foods in bulk, antibiotic resistance, multilocus sequence typing (MLST), multi-virulence-locus sequence typing (MVLST), epidemic clone (EC)

## Abstract

*Listeria monocytogenes* is an important foodborne pathogen causing public concern. A total of 3354 retail foods in bulk were sampled and screened for *L. monocytogenes*. Seventy-three (2.2%) samples including 21 ready-to-eat (RTE) foods and 52 raw foods were confirmed positive for *L. monocytogenes*. Sushi and salmon sashimi occupied the top two slots in RTE foods with relatively high presence rate of 12.9 and 6.9%, respectively. Meanwhile, *L. monocytogenes* was found to be distributed unequally in raw foods; the presence rates in raw meat (3.5%) and poultry (3.8%) were significantly higher than that in raw seafood (1.3%). Notably, *L. monocytogenes* was not detected in raw freshwater food. The *L*. *monocytogenes* isolates belonged to four serotypes, 1/2a, 1/2b, 1/2c, and 4b, with the most prevalent serotype being 1/2a (47.9%). Eighteen sequence types (STs) and eighteen virulence types (VTs) containing four newly assigned VTs (VT180, VT181, VT182, and VT183) were determined via multilocus sequence typing (MLST) and multi-virulence-locus sequence typing (MVLST). Among the 73 *L. monocytogenes* isolates, 23 (31.5%) belonged to epidemic clones (ECs) including ECI, ECIV, ECV, ECVI, ECVIII and ECXI among which ECV was predominant. Antibiotic susceptibility tests revealed a high resistance rate (11.0%) to tetracycline. Moreover, we identified the distribution patterns of virulence genes of four *Listeria* pathogenicity islands (LIPI) in *L. monocytogenes* isolates. *prfA*, *hly*, *plcA*, *plcB*, *mpl*, *actA* genes in LIPI-1 and *inlA*, *inlB*, *inlC*, *inlJ* genes in LIPI-2 were detected in approximately all *L. monocytogenes* isolates. The distribution of both LIPI-3 genes and LIPI-4 genes exhibited association with lineage and ST. LIPI-4 genes were present exclusively in ST87 isolates. Relatedness analysis revealed the absence of distinct association between STs, ECs, LIPI-3 and LIPI-4 distribution and specific food groups. This study provided fundamental data for Chinese food safety authorities to grasp the contamination status of *L. monocytogenes* in foods, assess the potential risk of this pathogen and further address the safety issue of retail foods in bulk in China.

## Introduction

*Listeria monocytogenes* is an important foodborne pathogen which can cause severe human listeriosis, particularly in older adults, newborns, pregnant women and immune-compromised individuals, resulting in septicemia, abortion, preterm delivery, stillbirth, meningitis, encephalomyelitis, or even death (Lomonaco et al., 2009; Lamont et al., 2011). In some cases, this bacterium’s capability to infect the central nervous system (CNS) of immunocompetent adults was also revealed (Guo and Liang, 2014; Giménez-Muñoz et al., 2015). The case-fatality rate of listeriosis attains 20–30% in several regions around the world (Centers for Disease Control and Prevention, 2017; European Food Safety Authority [EFSA] and European Centre for Disease Prevention and Control [ECDC], 2017; Food Standards Australia New Zealand, 2017; Li et al., 2018; Salama et al., 2018). Although not common, listeriosis shares a large proportion of the public health and economic burden resulting from foodborne pathogens. Almost 1500 listeriosis cases were reported annually in the United States, only accounting for 0.02% among major foodborne pathogens infection cases. However, 4% of hospitalization and 19% of deaths were caused by *L. monocytogenes* infection (Scallan et al., 2011).

*Listeria monocytogenes* extensively inhabits environments including surface soil, rivers, decaying vegetation, effluents from sewage treatments, wild animal intestines and domestic animal feces, due to its robust survival ability under various unfavorable conditions such as high salinity, low pH and low temperature (Donnelly, 2001; Vermeulen et al., 2007; Soni et al., 2013; Ribeiro and Destro, 2014; Ribeiro et al., 2014; Haley et al., 2015; Wang et al., 2017b). Wide distribution of *L. monocytogenes* offers opportunities to contaminate diverse foods, raw material and the environments of processing plants for food production (Heisick et al., 1989; Bula et al., 1995; Zhu et al., 2012; Wang et al., 2017b; Bergholz et al., 2018). Intake of contaminated food by *L. monocytogenes* has been widely considered to be the entry point of infection. In China, retail foods in bulk account for a large percentage of food consumption. Foods in bulk are defined as foods that are not divided into parts and/or packaged in separate units when sold in various sales locations. Compared with prepackaged foods, foods in bulk are prone to be directly exposed to the environment during the sales process, which makes it easier for them to be contaminated by pathogens. Surveying the prevalence, phenotypic and genotypic characteristics of *L. monocytogenes* in food products in bulk can provide valuable information on the distribution profiles of this pathogen, allowing Chinese food safety authorities to assess the potential risk of *L. monocytogenes* in bulk foods, and take appropriate hygiene measures to improve microbial safety. Epidemic clones (ECs) of *L. monocytogenes* are defined as genetically similar isolates descended from a common ancestor, involved in either a single large outbreak or geographically and temporally unrelated outbreaks (Kathariou, 2002; Cantinelli et al., 2013). Eleven known ECs including ECI - ECXI have been identified thus far (Chen et al., 2007, 2016; Lomonaco et al., 2013; Filipello et al., 2017). EC isolates were usually closely associated with listeriosis massive outbreaks (Haley et al., 2015). Since the first determined ECI outbreak in 1981, outbreaks of ECI were reported in western Switzerland during the time period 1983–1987, in California, United States in 1985, and in France in 1992 (Kathariou, 2002; Cantinelli et al., 2013). ECII caused an United States multistate hotdog outbreak and another turkey deli meat outbreak (Chen et al., 2011). In 2000, ECIII caused an United States outbreak attributed to intake of turkey deli meat. ECIV caused the United Kingdom and Ireland outbreak in 1987–1988, and an outbreak in Italy in 1997. ECV caused several major outbreaks occurring in the United Kingdom, Italy, Canada, and United States (Chen et al., 2011). ECVI and ECVII were newly defined in 2011, and these two epidemic clones led to 147 cases in 28 U.S. states, causing 33 deaths and one miscarriage (Lomonaco et al., 2013). ECVIII caused an United States milk chocolate outbreak in 1994, and an outbreak in Pennsylvania in 1987 (Chen et al., 2016). ECIX caused an United States multistate caramel apple outbreak in 2014–2015 (Chen et al., 2016). ECX caused a France pork rillettes outbreak in 1999–2000 (Chen et al., 2016). In 2012, ECXI caused an United States ricotta salata outbreak (Filipello et al., 2017). Clearly, ECs concealed in food products have the potential risk to cause *L. monocytogenes* infection, and even listeriosis outbreaks. Initially, ECs were confirmed based on the pulsed-field gel electrophoresis (PFGE) typing, ribotyping, and multilocus enzyme electrophoresis (MEE) (Kathariou, 2002; Kathariou et al., 2006). In subsequent studies, multi-virulence-locus sequence typing (MVLST) was developed and verified to discriminate ECs accurately (Chen et al., 2007; Lomonaco et al., 2013). Additionally, in some previous studies, the PCR assay was used to rapidly screen specific ECs markers to determine epidemic clones. However, these results were presumptive and should be confirmed using MVLST (Chen and Knabel, 2007; Knabel et al., 2012; Lomonaco et al., 2012). To date, ECs among *L. monocytogenes* strains isolated from foods in China were screened by means of PCR, and limited from ECI to ECIII (Wu et al., 2015, 2016). Accurate and comprehensive data for ECs distribution in foodborne *L. monocytogenes* isolates in China were still sparse. The aims of this study were (i) to reveal the prevalence of *L. monocytogenes* in retail foods in bulk sampled in Zhejiang Province, and (ii) to gain information on the phenotypic and genotypic characteristics, especially the EC profiles of *L. monocytogenes* isolates. Zhejiang Province is located on the southeast coast of China with a population of 57 million people. In 2018, the annual GDP (Gross Domestic Product) in Zhejiang Province was 81 billion USD. According to 2017 statistical data, the production of raw meat including pork, beef, and mutton was 869.900 tons, the total fishery production was 594.45 million tons and the number of poultry raised was 25,139,100 (Department of Agriculture of Zhejiang Province, 2017). The annual per capita raw meat, poultry and seafood consumption was 27.3, 11.5, and 26.4 kg respectively in 2017 (China Statistical Yearbook Committee, 2017).

## Materials and Methods

### Sampling and Isolation of *L. monocytogenes*

A total of 3354 retail foods in bulk including 1196 RTE foods and 2158 raw foods were sampled from supermarkets, farm fairs, fish markets, and restaurants in eleven geographic regions of Zhejiang Province, China from March 2016 to December 2016. The detailed information for food samples is shown in Table 1. Raw foods in this study were defined as not-ready-to-eat raw foods. During the sampling process, sterile containers were used to transfer representative units or portions of foods to the laboratory by aseptic practices. Frozen or chilled foods were transported in thermal insulated containers. Frozen foods including ice cream, frozen meat, frozen poultry and frozen seafood were stored at about −18°C and kept frozen prior to sampling. Chilled foods included chilled meat and chilled poultry stored at 0 – 5°C, as well as chilled seafood and salmon sashimi, were stored in crushed ice prior to sampling. Other categories of foods were stored at ambient temperature prior to sampling. Sample analysis was initiated as soon as the sample was received. All RTE foods except ice cream were sampled on the day of production. Ice cream was sampled during the shelf life period. Isolation of *L. monocytogenes* was carried out following the recommendation of the National Food Safety Standard of China – Food microbiological examination: *L. monocytogenes* (GB4789.30-2010; National Standard of the People’s Republic of China, 2010). Twenty-five grams of each sample was suspended in 225 mL *Listeria* enrichment broth LB1 (Huankai, Guangzhou, China) and homogenized for 2 min. The homogenate was incubated at 30°C for 24 h. 0.1 mL of each culture was transferred into 10 mL of *listeria* enrichment broth LB2 (Huankai, Guangzhou, China) and incubated at 30°C for 24 h, followed by spreading on CHROMagar Listeria plates (CHROMAgar, Paris, France). After incubation at 36°C for 48 h, five suspect colonies on each plate were selected for identification. If less than five colonies grew on any one plate, all suspect colonies were selected for identification. VITEK 2 compact system (Bio Mérieux, Lyon, France) and the following PCR method (Huang et al., 2007) were performed to identify each suspect colony. PCR was applied to discriminate *L. monocytogenes* from other *Listeria* species. *L. monocytogenes* strains EGDe (ATCC BAA-679) and 10403S were included as positive control strains for PCR. Serotyping was conducted adopting serum agglutination according to the manufacture’s instruction (Denka Seiken, Tokyo, Japan). All confirmed strains were stored at −70°C for further analysis.

**TABLE 1:** Prevalence of *L. monocytogenes* in different retail foods in bulk.

**Food category**	**No. of samples**	**No. of positive samples**	**Prevalence rate (%)**	**Sampling location (No. of samples)**
**Ready–to-eat food**				
Salad	192	6	3.1%	Shaoxing (66), Hangzhou (50), Jiaxing (30), Huzhou (26), Zhoushan (20)
Vegetable in sauce	52	1	1.9%	Wenzhou (14), Hangzhou (11), Shaoxing (10), Jinhua (9), Zhoushan (8)
Rice and noodle dishes	329	0	0%	Shaoxing (70), Hangzhou (68), Jiaxing (38), Lishui (36), Huzhou (26), Quzhou (26), Jinhua (25), Ningbo (20), Zhoushan (20)
Cooked meat	188	2	1.1%	Jinhua (34), Quzhou (30), Wenzhou (22), Hangzhou (20), Ningbo (20), Zhoushan (20), Jiaxing (16), Huzhou (14), Shaoxing (9), Lishui (3)
Sushi	62	8	12.9%	Hangzhou (22), Shaoxing (20), Huzhou (20)
Ice cream	30	0	0%	Hangzhou (16), Shaoxing (14)
Hamburger	112	0	0%	Hangzhou (28), Huzhou (20), Shaoxing (20), Lishui (18), Taizhou (16), Jiaxing (10)
Salmon sashimi	58	4	6.9%	Hangzhou (26), Shaoxing (12), Ningbo (10), Huzhou (10)
Cooked seafood	73	0	0%	Wenzhou (37), Taizhou (30), Jinhua (2), Zhoushan (2), Ningbo (1), Jiaxing (1)
Bakery product	100	0	0%	Jinhua (30), Hangzhou (26), Quzhou(18), Wenzhou (18), Zhoushan(8)
Subtotal	1196	21	1.8%	
**Raw meat**				
Fresh pork	342	11	3.2%	Shaoxing (70), Hangzhou (47), Wenzhou (42), Zhoushan (40), Huzhou (30), Jinhua (23), Jiaxing (22), Quzhou (20), Ningbo (16), Taizhou (16), Lishui (16)
Fresh beef	164	6	3.7%	Hangzhou (32), Wenzhou (24), Shaoxing (23), Huzhou (15), Zhoushan (13), Jinhua (12), Ningbo (11), Jiaxing (11), Lishui (11), Taizhou (8), Quzhou (4)
Fresh mutton	59	2	3.4%	Shaoxing (21), Lishui (9), Wenzhou (8), Huzhou (8), Zhoushan (5), Taizhou (5), Quzhou (3)
Chilled pork	43	1	2.3%	Hangzhou (24), Shaoxing (16), Huzhou (3)
Chilled beef	40	3	7.5%	Hangzhou (18), Shaoxing (15), Huzhou (7)
Chilled mutton	10	0	0%	Shaoxing (5), Hangzhou (4), Huzhou (1)
Frozen pork	6	0	0%	Hangzhou (3), Shaoxing (2), Jiaixng (1)
Frozen beef	8	1	12.5%	Hangzhou (5), Ningbo (1), Shaoxing (1), Jiaxing (1)
Frozen mutton	10	0	0%	Hangzhou (4), Shaoxing (3), Quzhou (2), Zhoushan (1)
Subtotal	682	24	3.5%	
**Raw poultry**				
Fresh chicken	344	11	3.2%	Hangzhou (55), Shaoxing (50), Huzhou (45), Wenzhou (30), Zhoushan (30), Jinhua (26), Ningbo (25), Quzhou (22), Taizhou (21), Lishui (20), Jiaxing (20)
Fresh duck	112	3	2.7%	Shaoxing (30), Lishui (12), Ningbo (11), Jiaxing (11), Wenzhou (10), Jinhua (10), Taizhou (8) Huzhou (7), Hangzhou (7), Quzhou (6)
Chilled chicken	31	3	9.7%	Shaoxing (10), Wenzhou (8), Huzhou (5), Quzhou (4), Jiaxing (2), Jinhua (2)
Chilled duck	10	0	0%	Wenzhou (4), Shaoxing (3), Huzhou (1), Jinhua (1), Quzhou (1)
Frozen chicken	44	3	6.8%	Hangzhou (25), Shaoxing (8), Quzhou (4), Jiaxing (3), Ningbo (2), Lishui (2)
Frozen Duck	14	1	7.1%	Shaoxing (8), Quzhou (3), Ningbo (2), Jinhua (1)
Subtotal	555	21	3.8%	
**Raw seafood**				
Fresh seafood	287	4	1.4%	Hangzhou (92), Wenzhou (54), Jiaxing (36), Zhoushan (33), Taizhou (32), Ningbo (22), Lishui (7), Jinhua (5), Huzhou (3), Quzhou (2), Shaoxing (1)
Chilled-fresh raw seafood	199	3	1.5%	Hangzhou (108), Zhoushan (35), Ningbo (21), Jiaxing (20), Jinhua (15)
Frozen raw seafood	39	0	0%	Hangzhou (29), Zhoushan (5), Jiaxing (4), Quzhou (1)
Subtotal	525	7	1.3%	
**Raw freshwater food**				
Fresh raw freshwater food	396	0	0%	Shaoxing (163), Jinhua (101), Lishui (44), Quzhou (44), Huzhou (42), Hangzhou (2)
Total	3354	73	2.2%	

### Multi-Locus Sequence Typing (MLST) Analysis

The total DNA of *L. monocytogenes* isolates was extracted using Bacterial DNA Kit (Omega, United States) according to the manufacturer’s protocol. DNA quality was tested by electrophoresis on 0.8% agarose gel and the concentration was determined by using Spectrophotometer NanoDrop 1000 (Thermo, United States). Prepared DNA was stored at −20°C for MLST, MVLST and virulence genes analysis. Seven housekeeping genes *abcZ*, *bglA*, *cat*, *dapE*, *dat*, *ldh*, *lhkA* were amplified according to the scheme advised in Institute Pasteur MLST database^1^ (Moura et al., 2016). Each amplicon was sequenced bidirectionally using 3730XL Genetic Analyser (Applied Biosystems, United States). Assembled nucleotide sequences were queried against Institute Pasteur MLST database. Sequence type (ST), clonal complex (CC) and lineage were then designated to each isolate. The phylogenic tree was constructed harnessing MEGA 5.1 (Tamura et al., 2011). Based on the concatenated sequence (3288 bp) of seven housekeeping genes, a neighbor-joining statistical method was employed. The number of Bootstrap replications was 1,000. The Genbank database accession numbers for *abcZ*, *bglA*, *cat*, *dapE*, *dat*, *ldh*, *lhkA* genes sequences of *L. monocytogenes* isolates in this study were MK368885 – MK368957, MK368812 – MK368884, MK368958 – MK369030, MK369031 – MK369103, MK369104 – MK369176, MK369177 – MK369249 and MK369250 – MK369322, respectively.

### Multi-Virulence-Locus Sequence Typing (MVLST) Analysis

Multi-virulence-locus sequence typing analysis was carried out following the method developed by Zhang et al. (2004). Six virulence genes including *prfA*, *inlB*, *inlC*, *dal*, *clpP*, and *lisR* were amplified and then sequenced. The nucleotide sequences were trimmed to the correct length and concatenated in a specific order as mentioned in the MVLST database^2^. Alignment was performed between the concatenated sequence of each isolate and the reference sequence of each virulence type (VT) available in the MVLST database by using BLAST 2.2.28+. VT and EC of each isolate were determined according to the alignment results. New VTs were designated by the MVLST database. The Genbank database accession numbers for *prfA*, *inlB*, *inlC*, *dal*, *clpP*, and *lisR* gene sequences of *L. monocytogenes* isolates in the current study were MK392393–MK392465, MK369469–MK369541, MK369542–MK369614, MK369396–MK369468, MK369323–MK369395, and MK369615–MK369687. The polymorphism of both MLST and MVLST genes was analyzed using DnaSP 5.10 software (Librado and Rozas, 2009). Analysis parameters included number of polymorphic sites, G + C content, *Ks* (the number of synonymous substitutions per synonymous site), *Ka* (the number of non-synonymous substitutions per non-synonymous site), π (average pairwise nucleotide difference per site) and Tajima’s *D* test for neutrality. Tajima’s *D* is a population genetic test statistic to distinguish between neutral evolution and non-neutral evolution of DNA sequences (Tajima, 1989).

### Antibiotic Susceptibility Tests

Antibiotic susceptibility of *L. monocytogenes* isolates was assayed using the broth micro-dilution minimum inhibitory concentrations (MICs) method according to the Clinical and Laboratory Standards Institute guidelines (Clinical and Laboratory Standards Institute, 2015). Breakpoints for Penicillin and Ampicillin were found in CLSI documents M45-A3 (Clinical and Laboratory Standards Institute, 2015). Since there is no relevant criteria for Erythromycin, Clindamycin, Quinupristin/dalfopristin, Vancomycin, Tetracycline, Gentamicin, Rifampin, Levofloxacin, Ciprofloxacin, Gatifloxacin, and Oxacillin, the susceptibility results for these antibiotics were interpreted based on the breakpoints of *Staphylococcus* spp (Clinical and Laboratory Standards Institute, 2017) as reported previously (Conter et al., 2009; Lungu et al., 2011; Chen et al., 2015; Khen et al., 2015; Tahoun et al., 2017). *L. monocytogenes* isolates resistant to three or more types of antibiotics belonging to different antibiotic classes were defined as multi-drug resistant (Magiorakos et al., 2012). *Escherichia coli* ATCC29522 and *S. aureus* ATCC29213 were used as quality control strains.

### Detection of Virulence Genes

The detailed information of primers used for virulence genes detection is shown in Table 2. Primers for *llsA*, *llsG*, *llsH*, *llsB*, *llsY*, *llsD*, *llsP* in LIPI-3 and all LIPI-4 genes were designed using PrimerPremier 5.0. The genome sequences of LIPI-3 positive *L. monocytogenes* strains including FSL N1-017, FSL R2-503, H7858 and NCTC 11994 were obtained from the NCBI Refseq genome database. Multiple alignment of nucleotide sequences of genes *llsA*, *llsG*, *llsH*, *llsB*, *llsY*, *llsD*, *llsP* mining from the above genomes were performed using clustal v1.83. The conserved region of each gene was used as a template for the primer’s design. Primers for LIPI-4 genes were designed according to the LIPI-4 sequence of *L. monocytogenes* strain LM09-00558 (Maury et al., 2016). PCR was conducted employing the performance system and conditions described previously (Zhang et al., 2017). The amplicons were analyzed with electrophoresis on 1% agarose gel. To validate the new primers for LIPI-3 and LIPI-4 genes, all associated positive PCR products were sequenced. The DNA sequences were analyzed using the BLAST algorithm on the website http://www.ncbi.nlm.nih.gov/BLAST.

**TABLE 2:** Primers used for *L. monocytogenes* virulence genes.

**Gene name or locus**	**Primer sequences (5′-3′)**	**Anneal temperature (°C)**	**Amplicon length (bp)**	**References**
**LIPI-1**				
*prfA*	F: 5′-AACGGGATAAAACCAAAACCA-3′	50	469	Zhang et al., 2004
	R: 5′-TGCGATGCCACTTGAATATC-3′			
*hly*	F: 5′-GTTAATGAACCTACAAGACCTTCC-3′	60	707	Wu et al., 2015
	R: 5′-ACCGTTCTCCACCATTCCCA-3′			
*plcA*	F: 5′-TCCCATTAGGTGGAAAAGCA-3′	50	840	Montero et al., 2015
	R: 5′-CGGGGAAGTCCATGATTAGA-3′			
*plcB*	F: 5′-CAGCTCCGCATGATATTGAC-3′	55	723	Montero et al., 2015
	R: 5′-CTGCCAAAGTTTGCTGTGAA-3′			
*mpl*	F: 5′-AAAGGTGGAGAAATTGATTCG-3′	55	450	Montero et al., 2015
	R: 5′-AGTGATCGTATTGTAGGCTGCTT-3′			
*actA*	F: 5′-AAACAGAAGAGCAGCCAAGC-3′	55	571	Montero et al., 2015
	R: 5′-TTCACTTCGGGATTTTCGTC-3′			
**LIPI-2**				
*inlA*	F: 5′-ACGAGTAACGGGACAAATGC-3′	55	800	Wieczorek et al., 2012
	R: 5′-CCCGACAGTGGTGCTAGATT-3′			
*inlB*	F: 5′-CATGGGAGAGTAACCCAACC-3′	55	367	Zhang et al., 2004
	R: 5′-GCGGTAACCCCTTTGTCATA-3′			
*inlC*	F: 5′-AATTCCCACAGGACACAACC-3′	55	517	Zhang et al., 2004
	R: 5′-CGGGAATGCAATTTTTCACTA-3′			
*inlJ*	F: 5′-TGTAACCCCGCTTACACACAGTT-3′	60	238	Wu et al., 2015
	R: 5′-AGCGGCTTGGCAGTCTAATA-3′			
**LIPI-3**				
*llsA*	F: 5′-ATGAATATTAAATCACAATCATCA-3′	50	150	This study
	R: 5′-TTACATTTTGGTTGCAGCAG-3′			
*llsG*	F: 5′-GAGACTGGGCTTACTTGC-3′	50	415	This study
	R: 5′-TACCTCCTGTTCACTGCTTG-3′			
*llsH*	F: 5′-ATGATGTTCGCTATGGTT-3′	45	421	This study
	R: 5′-ACATTCCTACTGGCATCA-3′			
*llsX*	F: 5′-TTATTGCATCAATTGTTCTAGGG-3′	55	200	Wieczorek et al., 2012
	R: 5′-CCCCTATAAACATCATGCTAGTG-3′			
*llsB*	F: 5′-TTACAATCAACCACCAGG-3′	45	334	This study
	R: 5′-AGTGAACCGAATGACAGA-3′			
*llsY*	F: 5′-ATTAGAATAGGAACGCAGAC-3′	50	581	This study
	R: 5′-TCATAGCACCCAGTTTCG-3′			
*llsD*	F: 5′-TATGGTGGTATGGAGGGT-3′	45	562	This study
	R: 5′-ATCACCCTGCTTATTTCA-3′			
*llsP*	F: 5′-TTTCCAGGTATGCTTCTT-3′	45	554	This study
	R: 5′-CAATTACGGTGGTTCTCA-3′			
**LIPI-4**				
*licC*	F: 5′-GGGATTCCGAAACTACCT-3′	50	736	This study
	R: 5′-CGAGTGCTCCTGTAACCC-3′			
*licB*	F: 5′-ATTGCGGCATCTGAGAAA-3′	55	232	This study
	R: 5′-CAGCGATTAGAATTGGTACTGC-3′			
*licA*	F: 5′-GCCTCTTCCTCGTTTCTA-3′	45	227	This study
	R: 5′-GACTTAACTAAATCGCAGTA-3′			
Lm900558-70012	F: 5′-TGGTAACAATGCCTGCTT-3′	50	369	This study
	R: 5′-GCTGAAAGCCCACTGTAT-3′			
Lm900558-70013	F: 5′-TATTCAGTGGTTACGAGGCT-3′	50	403	This study
	R: 5′-CTCCGCCGAAATCTGGTA-3′			
*glvA*	F: 5′-TTACTATTGCTGGCGGAGGA-3′	55	847	This study
	R: 5′-TGCTCACGACCATCCATT-3′			

### Statistical Analysis

Chi-square analysis or Fisher’s exact test was performed to determine if a significant difference in the prevalence and serotype distribution of *L. monocytogenes* among different food groups could be found. The significance level was set at a *p*-value of <0.05. All analyses were performed using the SPSS v 21.0 software package. Association between food groups and genotyping data, antibiotic resistance profiles, and *Listeria* pathogenicity islands (LIPI) distribution was calculated based on the Gini coefficient (Henri et al., 2016) using the Stata software. A coefficient value of smaller than 0.4 reveals no association between genotypes, antibiotic resistance profiles or LIPI distribution and food types, a value between 0.4 and 0.6 indicates moderate association, and a value greater than 0.6 indicates unequal dispersion of genotypes, antibiotic resistance profiles or LIPI within food groups.

## Results and Discussion

### Prevalence of *L. monocytogenes* in Retail Foods in Bulk

The prevalence of *L. monocytogene*s in various analyzed food samples was shown in Table 1. Seventy-three out of 3354 samples were confirmed to be *L. monocytogenes* positive with the total rate of 2.2%. Among them, 21 strains (1.8%) were isolated from 1196 ready-to-eat (RTE) foods and 52 strains (2.4%) were isolated from 2158 raw foods. No statistically significant difference in the prevalence of *L. monocytogenes* was present between RTE foods and raw foods (χ^2^ = 1.545, *p* > 0.05). RTE foods contaminated with pathogenic microorganisms have been widely considered to be the major source of foodborne pathogen infections due to the absence of further cooking, baking or pasteurizing processes prior to consumption (European Food Safety Authority [EFSA], 2013; Pouillot et al., 2015; Luchansky et al., 2017). Here *L. monocytogenes* was isolated in five RTE food categories including sushi, salmon sashimi, salad, vegetables in sauce and cooked meat. The occurrence of *L. monocytogenes* in sushi and salmon sashimi exhibited the top two highest rates (12.9 and 6.9%) among RTE foods and significantly higher than that in all screened RTE foods (1.8%) (χ^2^ = 27.762, *p* = 0; χ^2^ = 5.082, *p* = 0.024) and cooked meat (1.1%) (χ^2^ = 14.076, *p* = 0; χ^2^ = 4.123, *p* = 0.042). Relatively high presence rates of *L. monocytogenes* in sushi and salmon sashimi poses a potential risk of causing human *L. monocytogenes* infection. RTE meat products are considered one of the important food sources of human *L. monocytogenes* infection throughout the world (European Food Safety Authority [EFSA], 2013; Iannetti et al., 2016; Raheem, 2016). According to our data, the prevalence rate of *L. monocytogenes* in cooked meat was close to that in prepackaged heated meat at the end of its shelf-life in the European Union as well as meat products in Italy (2.07 and 1.66% respectively) (European Food Safety Authority [EFSA], 2013; Iannetti et al., 2016).

*Listeria monocytogenes* was distributed unequally in raw foods. Presence rates in raw meat (3.5%) and raw poultry (3.8%) were significantly higher than that in raw seafood (1.3%) (χ^2^ = 5.663, *p* = 0.017; χ^2^ = 6.415, *p* = 0.011). It is worthy to note that *L. monocytogenes* was not detected in 396 samples of raw freshwater food. We further analyzed if rates of *L. monocytogenes* in raw foods under different storage conditions were different. The results did not show a significant difference among fresh, chilled and frozen foods. Due to the common eating habit in China of cooking foods completely, raw foods were not predicted to have a high risk of causing *L. monocytogenes* infection in China. However, transfer of *L. monocytogenes* might occur from contaminated raw foods to RTE foods during the process of storage after purchase. In particular, cross contamination may be more likely to occur due to lack of tight packaging for foods in bulk.

### Serotyping

**FIGURE 1:**
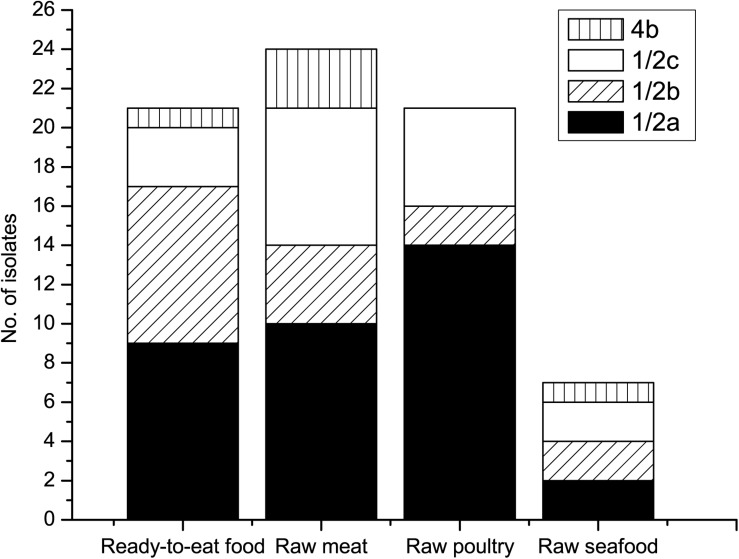
Serotypes of *L. monocytogenes* isolates from various foods.

Four different serotypes, 1/2a, 1/2b, 1/2c, and 4b were identified in the 73 *L. monocytogenes* isolates (Figure 1). 1/2a was the most prevalent serotype, accounting for 47.9% of the identified isolates. Serotype 1/2a strains have exhibited extensive distribution in various foods around the world, which might be due to superior adaptability in different environments compared to other serotype strains (Korsak et al., 2012; Schmitz-Esser et al., 2015; Wu et al., 2015). Serotypes 1/2b and 1/2c had similar presence rates at 21.9 and 23.3%, respectively. Among the 73 isolated strains, only five (6.8%) were confirmed as serotype 4b. Serotype distribution difference among different food groups was analyzed based on Chi-square or Fisher analysis, as appropriate. The prevalence of serotype 1/2a in raw poultry was significantly higher than that in raw seafood (χ^2^ = 8.478, *p* = 0.004) and RTE food (χ^2^ = 6.025, *p* = 0.014). Meanwhile, there was no obvious distribution difference of serotypes 1/2b, 1/2c, and 4b among different food groups (*p* > 0.05). The current study showed that a total of 52 (71.2%) isolates were grouped into 1/2a, 1/2b, and 4b serotypes, which were the main causative agents of clinical cases worldwide and were responsible for 98% of listeriosis outbreaks (Buchrieser et al., 1993; Raheem, 2016). Among all the confirmed serotypes, no serotypes 3a, 3b, 3c, 4a, 4c, 4d, 4e, or 7 were detected, which was consistent with a previous report (Wu et al., 2015) in China indicating that those serotypes were rarely identified from food and clinical samples.

### MLST Analysis

Eighteen different sequence types (STs) were classified among all 73 *L. monocytogenes* isolates, which were further assigned to sixteen clonal complexes (CCs) and one singleton by using MLST analysis, with the Simpson’s index of diversity (DI) at 0.892 (Table 3). Among the 18 STs, ST9 (23.3%) was predominant, followed by ST155 (16.4%), ST8 (12.3%), and ST121 (9.6%). Many studies have shown that hypervirulence exists in *L. monocytogenes* with different STs. Although ST9, ST155, ST8, and ST121 were all revealed to be involved in *L. monocytogenes* infection, they tended to be isolated from food products and food processing environments. Only a small number of clinical cases were induced by these ST types (Ebner et al., 2015; Schmitz-Esser et al., 2015; Wu et al., 2016; Maury et al., 2016; Wang et al., 2018). For instance, while ST9 and ST121 were two major STs among *L. monocytogenes* foodborne isolates during 1999–2014 in France, ST121 included the fewest clinical isolates (Henri et al., 2016). ST1 and ST2 clones have been verified to be strongly associated with clinical origin particularly in human CNS or maternal-neonatal (MN) listeriosis cases (Maury et al., 2016). According to our data, one isolate from fresh pork and three isolates from sushi, chilled beef and chilled seafood were genotyped into ST1 and ST2, respectively. Although the presence rates (1.4 and 4.1%) were relatively low, the existence of ST1 and ST2 in foods, particularly in RTE foods, poses a potential risk on food safety due to their high virulence. ST87 clones were detected in four samples including two RTE foods. ST87 strains were scarcely isolated from food, environmental or clinical samples in North America, South America, Europe, and Australia (Maury et al., 2016). In contrast, ST87 was the predominant ST in clinical *L. monocytogenes* isolates and closely related to CNS and MN infection in China (Wang et al., 2015, 2018; Wu et al., 2016).

**TABLE 3:** MLST characteristics of *L. monocytogenes* isolates.

**STs**	**Clonal complex (CC)**	**Lineage**	**Serotype**	**No. of isolates (%)**	**Strain origin**
ST1	CC1	I	4b	1 (1.4)	Fresh pork (1)^*^
ST2	CC2	I	4b	3 (4.1)	Sushi (1); Chilled beef (1); Chilled seafood (1)
ST3	CC3	I	1/2b	4 (5.5)	Sushi (3); Fresh chicken (1)
ST5	CC5	I	1/2b	4 (5.5)	Cooked meat (1); Frozen beef (1); Vegetables in sauce (1); Fresh seafood (1)
ST8	CC8	II	1/2a	9 (12.3)	Fresh chicken (3); Frozen chicken (1); Fresh duck (1); Fresh pork (2); Fresh mutton (1); Chilled seafood (1)
ST9	CC9	II	1/2c	17 (23.3)	Fresh chicken (3); Chilled chicken (1); Frozen chicken (1); Fresh beef (3); Chilled beef (1); Salad (3); Fresh pork (3); Chilled seafood (1); Fresh seafood (1)
ST31	CC31	II	1/2a	3 (4.1)	Sushi (2); Salmon sashimi (1)
ST87	CC87	I	1/2b	4 (5.5)	Salmon sashimi (1); Frozen duck (1); Cooked meat (1); Fresh seafood (1)
ST101	CC101	II	1/2a	1 (1.4)	Sushi (1)
ST121	CC121	II	1/2a	7 (9.6)	Fresh chicken (2); Chilled chicken (2); Frozen chicken (1); Fresh duck (1); Chilled pork (1)
ST145	CC2	I	4b	1 (1.4)	Chilled beef (1)
ST155	CC155	II	1/2a	12 (16.4)	Salmon sashimi (1); Fresh duck (1); Fresh beef (2); Salad (3); Fresh pork (3); Fresh mutton (1); Fresh seafood (1)
ST204	CC204	II	1/2a	1 (1.4)	Sushi (1)
ST224	CC224	I	1/2b	1 (1.4)	Fresh beef (1)
ST307	CC307	II	1/2a	2 (2.7)	Fresh chicken (2)
ST330	CC288	I	1/2b	1 (1.4)	Fresh pork (1)
ST426	CC426	I	1/2b	1 (1.4)	Salmon sashimi (1)
ST1047	Singleton 1047	I	1/2b	1 (1.4)	Fresh pork (1)

^*^Number in parentheses indicates the number of strains isolated from the given food category.

### MVLST and EC Analysis

**FIGURE 2:**
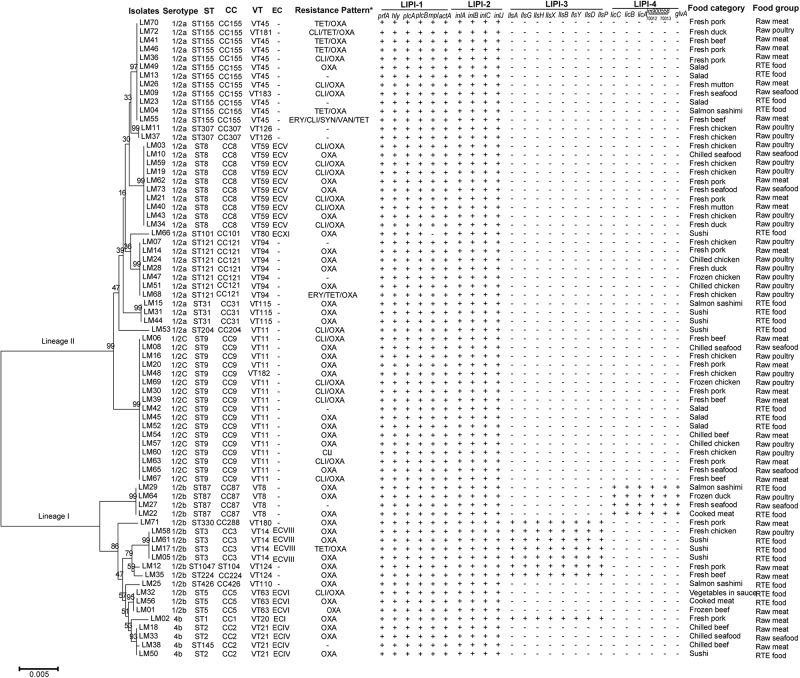
Characteristics of *Listeria monocytogenes* strains isolated from retail foods in bulk in Zhejiang Province, China. The neighbor-joining phylogeny tree was computed by MEGA 5.1 with Bootstrap replications number set 1,000. ^*^: TET, tetracycline; OXA, oxacillin; CLI, clindamycin; ERY, erythromycin; SYN, quinupristin/dalfopristin; VAN, vancomycin.

A total of eighteen VTs including four newly assigned VTs (VT180, VT181, VT182, and VT183) were determined in all *L. monocytogenes* isolates (Figure 2). Among 73 strains, 23 (31.5%) of them were epidemic clones including ECI, ECIV, ECV, ECVI, ECVIII, and ECXI, which is different from previous reports indicating that ECI and ECIII are the only detected ECs in China (Wu et al., 2015, 2016). ECV was most common; 10 out of 23 (43.5%) EC isolates were classified into this group.

To explore the polymorphisms of MLST and MVLST genes in *L. monocytogenes* population investigated in this study, the number of polymorphic sites, *Ka/Ks* ratio, π and Tajima’s *D* of associated genes in 73 *L. monocytogenes* isolates were calculated (Table 4). Seven MLST genes contained a total of 151 polymorphic sites (4.6%, range 4.2–12.1% per gene). Six MVLST virulence genes contained a total of 150 polymorphic sites (5.7%, range 1.1–14.2% per gene). *Dat* and *dal* had the two highest percentages of polymorphic sites, respectively. *Ka/Ks* of all MLST genes as well as MVLST genes *dal*, *inlB*, *inlC*, *prfA* were less than one, indicating these genes evolved under purifying (negative) selection. Meanwhile, *Ka/Ks* of *clpP* was 3.551, which indicated *clpP* was under positive selection during the course of genetic evolution (Zhang and Yu, 2006). Through comparison analysis of *Ka/Ks* between our data and previous reports (Cantinelli et al., 2013; Wu et al., 2016), MLST genes in different *L. monocytogenes* populations demonstrated similar selective strength. However, the selective strength for MVLST genes presented to be variable in *L. monocytogenes* populations from different ecological niches. For instance, *inlB* in *L. monocytogenes* outbreak strains in France was under neutral selection. Meanwhile, *clpP* was under purifying (negative) selection (Cantinelli et al., 2013). The average nucleotide diversity was close between MLST genes and MVLST genes, with π equaling 1.8% (range 1.0–5.6% per gene) and 2.1% (range 0.3–6.1% per gene) respectively. Tajima’s *D* test illustrated that *abcZ*, *bglA*, *ldh*, *lhkA*, *inlB*, *inlC*, *lisR*, and *prfA* evolved neutrally, whereas *cat*, *dapE*, *dat*, *clpP*, *dal* evolved under balancing selection.

**TABLE 4:** Polymorphism of MLST and MVLST genes in *L. monocytogenes* isolates.

	**Gene**	**Size (bp)**	**GC content (%)**	**No. (%) polymorphic sites**	****Ks****	****Ka****	****Ka**/**Ks****	**π(%)**	**Tajima’s **D****
MLST	**abcZ**	537	37.5	31 (5.8)	0.07796	0.00087	0.011	1.759	1.44291
	**bglA**	399	40.7	21 (5.3)	0.04531	0.00069	0.015	1.037	−0.15876
	**cat**	486	41.1	22 (4.5)	0.07746	0.00135	0.017	1.795	2.63086^*^
	**dapE**	462	43.8	36 (7.8)	0.13208	0.00712	0.054	3.413	2.71695^∗∗^
	**dat**	471	36.5	57 (12.1)	0.20685	0.01232	0.060	5.587	3.08223^∗∗^
	**ldh**	453	43.4	19 (4.2)	0.06103	0.00008	0.001	1.396	1.76974
	**lhkA**	480	37.3	20 (4.2)	0.05357	0.00239	0.045	1.377	1.72851
	Concatenate	3288	40.4	151 (4.6)	0.07257	0.00214	0.030	1.762	1.93145
MVLST	**clpP**	416	39.7	14 (3.3)	0.00442	0.01569	3.551	1.327	2.51168^*^
	**dal**	438	40.9	62 (14.2)	0.22553	0.01484	0.066	6.124	2.48712^*^
	**inlB**	433	40.3	35 (8.1)	0.09282	0.00720	0.078	2.720	1.59339
	**inlC**	415	31.5	15 (3.6)	0.04001	0.00407	0.102	1.192	1.67372
	**lisR**	444	38.6	5 (1.1)	0.00000	0.00416	–	0.331	0.94608
	**PrfA**	469	34.5	19 (4.1)	0.05663	0.00000	0.000	1.233	1.26807
	Concatenate	2615	37.6	150 (5.7)	0.00000	0.00000	0.000	2.113	2.18230^*^

Ks, the number of synonymous substitutions per synonymous site; Ka, the number of non-synonymous substitutions per non-synonymous site. π, average pairwise nucleotide difference per site. ^*^: Statistic difference of *p* < 0.05; ^∗∗^: Statistic difference of *p* < 0.01.

### Antibiotic Susceptibility of *L. monocytogenes* Isolates

The susceptibilities of *L. monocytogenes* isolates to thirteen antibiotics were shown in Table 5. All 73 strains were susceptible to penicillin, ampicillin, gentamicin and rifampin. More than 95% of strains were susceptible to erythromycin (97.3%), quinupristin/dalfopristin (98.6%), vancomycin (98.6%), levofloxacin (98.6%) and ciprofloxacin (95.9%). The results were similar to previous reports from China, the United States, Ireland, and Poland (Wieczorek et al., 2012; Shen et al., 2013; Chen et al., 2015; Khen et al., 2015; Wieczorek and Osek, 2017) and consistent with the general thought that the *Listeria* genus is naturally susceptible to ampicillin, penicillin, gentamicin and erythromycin, which are usually active against Gram-positive bacteria (Wang et al., 2013; Chen et al., 2015; Wu et al., 2015, 2016). On the other hand, *L. monocytogenes* strains isolated from raw milk, milk equipment and farm workers in Egypt showed lower susceptibility rates to gentamicin (19.0%), rifampin (0%) and ciprofloxacin (42.9%) (Tahoun et al., 2017). The most prevalent detected antibiotic resistance type was resistance to oxacillin. 86.3% of *L. monocytogenes* isolates were resistant to this antibiotic. Compared with previously reported resistance rates (1.0–4.5%) to tetracycline of *L. monocytogenes* isolated from various types of raw foods in China and beef chain in Ireland (Wieczorek et al., 2012; Shen et al., 2013; Wang et al., 2013; Chen et al., 2015; Khen et al., 2015), a much higher resistance rate of 11.0% to tetracycline was observed among our isolates. In the reference listeriosis therapy scheme, ampicillin or penicillin G combined with gentamicin was recommended as the treatment of choice. Meanwhile, vancomycin, trimethoprim-sulfamethoxazole and erythromycin were usually used as alternatives, especially for pregnant women (Hof, 2004). Both our results and previous reports (Rodas-Suarez et al., 2006; Morvan et al., 2010; Lungu et al., 2011; Wang et al., 2013; Chen et al., 2015; Tahoun et al., 2017) showed relatively low resistance rates to ampicillin, penicillin, gentamicin, vancomycin, trimethoprim-sulfamethoxazole and erythromycin, which revealed that the antibiotic treatment might be efficient for most of the *L. monocytogenes* strains. Including three (4.1%) multidrug-resistant strains, resistant to Erythromycin/Clindamycin/Quinupristin/dalfopristin/ Vancomycin/Tetracycline, Clindamycin/Tetracycline/Oxacillin and Erythromycin/Tetracycline/Oxacillin, respectively, all 73 isolates were grouped into seven antibiotic resistance patterns. It is worthy to note that one strain was identified as resistant to six types of antibiotics (Figure 2).

**TABLE 5:** Antibiotic susceptibility of *L. monocytogenes* isolates.

**Antimicrobial class**	**Antimicrobial agents**	**MIC (μg/ml) Interpretive Criteria**			**No. of isolates (%)**		
		**Susceptible**	**Intermediate**	**Resistant**	**Susceptible**	**Intermediate**	**Resistant**
Penicillins and β-Lactam/β-Lactamase Inhibitor Combinants	Penicillin^a^	≤2	–	–	73 (100%)	0 (0%)	0 (0%)
	Ampicillin^a^	≤2	–	–	73 (100%)	0 (0%)	0 (0%)
	Oxacillin^b^	≤2		≥4	10 (13.7%)	0 (0%)	63 (86.3%)
Macrolides	Erythromycin^b^	≤0.5	1–4	≥8	71 (97.3%)	0 (0%)	2 (2.7%)
Lincosamide	Clindamycin^b^	≤0.5	1–2	≥4	2 (2.7%)	51 (69.9%)	20 (27.4%)
Streptogramins	Quinupristin/dalfopristin^b^	≤1	2	≥4	72 (98.6%)	0 (0%)	1 (1.4%)
Glycopeptides	Vancomycin^b^	≤2	4-8	≥16	72 (98.6%)	0 (0%)	1 (1.4%)
Tetracyclines	Tetracycline^b^	≤4	8	≥16	65 (89.0%)	0 (0%)	8 (11.0%)
Aminoglycosides	Gentamicin^b^	≤4	8	≥16	73 (100%)	0 (0%)	0 (0%)
Ansamycins	Rifampin^b^	≤1	2	≥4	73 (100%)	0 (0%)	0 (0%)
Quinolones	Levofloxacin^b^	≤1	2	≥4	72 (98.6%)	1 (1.4%)	0 (0%)
	Ciprofloxacin^b^	≤1	2	≥4	70 (95.9%)	3 (4.1%)	0 (0%)
	Gatifloxacin^b^	≤0.5	1	≥2	0 (0%)	73 (100%)	0 (0%)

*^*a*^Breakpoints for *Listeria monocytogenes*. ^*b*^Breakpoints for Staphylococcus spp.*

### Virulence Genes Profile of *L. monocytogenes* Isolates

The molecular determinants of *L. monocytogenes* virulence has been investigated for many years (Tilney and Portnoy, 1989; Cossart, 2011). Four *Listeria* pathogenicity islands (LIPI) have been verified thus far, which are involved in invasion, survival and colonization of *L. monocytogenes* in host tissues. LIPI-1 contains six genes including *hly*, *prfA*, *plcA*, *plcB*, *mpl*, and *actA* (Hamon and Cossart, 2011; Travier and Lecuit, 2014; Mitchell et al., 2015; Rupp et al., 2015; Wang et al., 2017a; Hadjilouka et al., 2018; Poimenidou et al., 2018). LIPI-2 encodes a series of internalin family proteins which interact with the molecular cell surface and are essential for host cell adherence and virulence (McGann et al., 2007). LIPI-3 contributes to the expression of listeriolysin S (LLS), which is a post-translationally modified hemolytic peptide acting as a bacteriocin to alter the host intestinal microbiota, and plays an important role in the survival of *L. monocytogenes* in polymorphonucleocytes (PMNs) and virulence in the murine model (Cotter et al., 2008; Quereda et al., 2016). LIPI-4 encodes a cellobiose-family phosphotransfer system (PTS) and is involved in neural and placental infection (Maury et al., 2016). The distribution of virulence genes of these LIPIs in *L. monocytogenes* isolates were tested in this study (Figure 2). LIPI-1 genes were detected in approximately all *L. monocytogenes* isolates except one ST101 strain isolated from sushi, in which *mpl* was absent. *inlA*, *inlB*, *inlC* and *inlJ* of LIPI-2 existed in all 73 strains. 11.0% of isolates harbored all LIPI-3 genes and four isolates (5.5%) were determined LIPI-4 genes positive.

Remarkably, the distribution of both LIPI-3 and LIPI-4 exhibited apparent association with *L. monocytogenes* lineage and ST. LIPI-3 positive strains belonged to ST1, ST3, ST224, ST330, and ST1047; these STs were grouped into lineage I, in accordance with the finding that LIPI-3 was identified exclusively in a subset of lineage I (Clayton et al., 2014; Quereda et al., 2016). LIPI-4 was verified to exist uniquely in CC4 *L. monocytogenes* and closely linked to high virulence in CNS and MN listeriosis (Maury et al., 2016). A subsequent study pointed out that ST619, CC87 strains carried LIPI-4 fragment *ptsA* (Wang et al., 2018). In this study, we detected six genes encoding PTS sugar transporter subunit EIIC (*licC*), EIIB (*licB*), EIIA (*licA*), PTS systems associated protein (gene locus in Genbank, lm900558-70012), transcriptional antiterminator (gene locus in Genbank, lm900558-70013) and maltose-6′-phosphate-glucosidase (*glvA*) (Maury et al., 2016) of LIPI-4 in all ST87 isolates, which confirmed that this *L. monocytogenes* group carried all LIPI -4 genes. Meanwhile, the test of virulence genes was based on the PCR method employing target gene specific primers. There are some disadvantages to this method, including (i) polymorphism might exist in the primer annealing regions in the genomes of certain *L. monocytogenes* strains, leading to invalid or inefficient binding to primers and then false negative results, (ii) specificity of primers is not sufficient to avoid amplification of non-target regions. In recent years, whole genome sequencing (WGS) was utilized in many studies on *L. monocytogenes*, including the determination of virulence genes profiles (Maury et al., 2016; Fox et al., 2017; Rychli et al., 2017; Pightling et al., 2018), which can avoid false negative or positive results significantly. Genetic diversity of virulence genes can be further analyzed based on the nucleotide sequence.

Additionally, Gini coefficient analysis did not find an association between STs, ECs, LIPI-3 and LIPI-4 distribution of *L. monocytogenes* isolates and food groups. Values of the coefficient for eighteen STs ranged from 0.0 to 0.38. Seven ECs ranged from 0.0 to 0.25: 0.25 for LIPI-3 and 0.17 for LIPI-4, which indicated that STs, ECs, LIPI-3, and LIPI-4 were distributed uniformly within the four food groups: RTE food, raw meat, raw poultry and raw seafood. Meanwhile, moderate association was demonstrated between antibiotic resistance to oxacillin and raw poultry (coefficient value of 0.46), indicating that *L. monocytogenes* isolated from RTE food and raw poultry tend to be resistant to oxacillin.

## Conclusion

In summary, a comprehensive study of prevalence and characteristics of *L. monocytogenes* isolated from retail foods in bulk in Zhejiang Province, China was performed. Both RTE foods and raw foods were included, showing a wide range of food categories. The potential risk of causing human *L. monocytogenes* infection by certain foods with relatively high contamination rates, including sushi and salmon sashimi, should arouse public concern. Distribution differences of serotype 1/2a among different food groups revealed that this serotype of *L. monocytogenes* might have specific ecological niches. To the best of our knowledge, this is the first time the distribution of ECs (ECI-ECXI) has been investigated in foods sampled in China. Furthermore, the discovery of multidrug-resistant strains and the particularly high resistance rate (11.0%) to tetracycline among *L. monocytogenes* isolates indicates a potential public health problem. According to our relatedness analysis, ECs and LIPI-3 or LIPI-4 positive isolates were distributed equally among various food groups. The present study provides initial data for Chinese food safety authorities to address the issue of microbial safety of retail bulk foods in China. One recommendation is to strengthen the monitoring of retail foods in bulk with relatively high detection rates of *L. monocytogenes*, including sushi and salmon sashimi. In addition, the public must recognize the potential risks of certain foods with high contamination rate of *L. monocytogenes*, and a national standard should be developed for the detection limit of *L. monocytogenes* for bulk foods with high risk of causing *L. monocytogenes* infection.

## Author Contributions

LZ, LM, and YZ designed the experiments. YZ, LZ, HC, JC, JZ, ZZ, YY, and ZX carried out the experiments. YZ and SD analyzed the experimental results and wrote the manuscript.

## Conflict of Interest Statement

The authors declare that the research was conducted in the absence of any commercial or financial relationships that could be construed as a potential conflict of interest.

## Footnotes

1 https://bigsdb.pasteur.fr/listeria/listeria.html

2 https://sites.google.com/site/mvlstdatabase/home
